# Health and quality of life among women after participation in a CBPR-informed physical activity intervention: with a pandemic perspective

**DOI:** 10.1038/s41598-023-45239-4

**Published:** 2023-10-20

**Authors:** Rathi Ramji, Margareta Rämgård, Elisabeth Carlson, Sergey Shleev, Eman Awad, Stefan Cirovic, Anders Kottorp

**Affiliations:** 1 Department of Care Science, Faculty of Health and Society, Malmö University, Jan Waldenströms Gata 25, 20506 Malmö, Sweden; 2 Department of Biomedical Science, Faculty of Health and Society, Malmö University, Jan Waldenströms Gata 25, 20506 Malmö, Sweden

**Keywords:** Risk factors, Health care

## Abstract

The lack of culturally and contextually oriented interventions promoting physical activity (PA) has led to increased physical inactivity among women living in disadvantaged neighbourhoods in Sweden. In this study one such intervention informed by community-based participatory research (CBPR) has been evaluated among 34 women from a disadvantaged neighbourhood before and during COVID-19. Health-related quality of life (HRQOL), behavioural and biomedical outcomes were assessed directly prior and post-intervention, followed by evaluations at 6-months and 18-months follow-up during COVID-19. The results revealed that HRQOL, particularly psychological, social, and environmental health significantly increased post-intervention compared to prior to intervention but reversed back at 6-months follow-up. Perceived health satisfaction and environmental health increased at 18-months follow-up during COVID-19. Participation in PA improved post-intervention and at 6-months follow-up. Everyday activities and fruit and vegetable intake continued to increase through all timepoints. Systolic blood pressure significantly decreased post-intervention and 6-months follow-up; blood flow rate increased significantly at all timepoints. Overall, the findings underscores the potential effectiveness of CBPR approaches in promoting and sustaining healthy lifestyles, even during acute situations such as the COVID-19. It may even serve as a future model for promoting health and addressing health disparities in similar groups.

## Introduction

Physical activity is an essential behavioural factor that contributes to overall health and well-being of individuals. Recent research has even shown physical activity as a therapeutic strategy to mitigate COVID-19 since it induces anti-inflammatory response in the tissues and bloodstream which inhibits the immunosuppressive mechanism of the virus restricting the spread of infection while also alleviating related symptoms of depression and anxiety^1–5^. Despite the fact that the World Health Organization (WHO) recommends 150 min of moderate intensity aerobic activity per week for adults aged 18–64 years, nearly 30% of the global population over the age of 15 years are physically inactive. Thus, physical inactivity has been regarded as a widespread global pandemic^6,7^. While it is known that physical activity levels seem to differ by age, gender, and disability^8^, research suggests a social gradient associated with it. This in particular is related to differences in factors such as social and physical place in which individuals thrive, including access to health care system which may influence individual choice of lifestyle and thereby their health and quality of life. Lack of access to a healthy lifestyle owing to socioeconomic factors has contributed to increasing health inequalities given the rapid rise in chronic diseases which also exerts a substantial economic burden on health systems of the countries in the world.

This seems also true even in a welfare state as Sweden where a vast majority (65%) of the Swedish adult population follow the WHO recommendation for physical activity^9,10^. A Swedish population study mapping population risk factors for diabetes has identified disparities in physical activity associated with socio economic conditions and neighbourhood deprivation^11^. Residents living in a low socio-economic or disadvantaged neighbourhood in larger cities in Sweden, exhibited low fitness levels due to prolonged sitting and lack of access to physical activity, such as sport or training^12,13^. Problems with integration in these communities has led to low social contact, segregation, and mental ill-health^14,15^. These communities also face issues such as unemployment and those few that are employed tend to have poor work-life balance leading to lack of time for self-care or physical activity^15^.

Population level studies from several regions in Sweden show that women predominantly from multicultural background living in socially disadvantaged neighbourhoods were often physically inactive owing to lack of culturally and contextually oriented efforts to promote physical activity^16,17^. Women from these kind of neighbourhoods frequently experience more challenges to engage in physical activity than their male counterparts owing to being financially dependent to their spouse also related to cultural aspects which may hinder their possibility to access physical activity facilities. Previous research has also shown that the rate of chronic disease seemed higher particularly among these women due to their lack of access to knowledge regarding behavioural risk factors and the lack of access to health care services owing to financial and language barriers, resulting in delayed access to primary care, late diagnosis, and treatment^10,18–20^. Despite having the greatest health risks these women are often disproportionately exposed to health promotional initiatives especially those offered at a population level^21,22^.

In the Nordic context, the municipality and the regional authorities are responsible for public health related activities at the local level. However, community-based preventive and promotive initiatives by the health care sector which is adapted to the citizens’ needs and is built based on constructive dialogues with the citizens are sparse. At the same time, there are fewer opportunities for poor migrants given that the investment in the public sector has been reduced considerably over the years, creating a health gap. Aside from this COVID-19 pandemic which predominantly affected citizens in disadvantaged neighbourhoods has led to a health crisis further accelerating the physical inactivity pandemic which the public health authorities have failed to address for over two decades^23,24^. Despite the fewer COVID-19 restrictions in Sweden, studies show that negative changes in lifestyle including physical inactivity, which was significantly higher among women from socially disadvantaged neighbourhood as they spent more time in sitting at home, and had higher odds of mental ill-health^25^.

Experiences drawn from previous studies in similar contexts suggest that citizens often require support in form of tailored community health programmes which are contextually responsive and also help to deal with the complexities of existing and also newly emerging diseases^26–28^. Further evidence suggests that tailored initiatives should also strive to promote integration to society so public actors such as the social services and health care^29^.

Community-based participatory research (CBPR) is an approach where citizens from the communities act as partners and are active in conceptualizing a research problem to final dissemination and evaluation of the programme together with academic researchers and other stakeholders^30–33^. CBPR interventions has also been successful in protecting health and well-being during acute situations such as pandemics and natural disasters given that the equitable partnership between the community and the academicians helps mitigate stress and promotes resilience and recovery^27,28,34–36^. Previous studies suggest that behavioural interventions are more likely to be sustainable when interventions are developed together with the community through a community academic partnership (CAP) built on trust. A CBPR approach assumes that the communities possess invaluable experiential knowledge that is often not taken into consideration when developing traditional behavioural interventions^37,38^. It is therefore important to take the local context where the communities thrive into consideration when creating interventions targeting behavioural change. This also involves sharing of power with the community in reflective dialogues while discussing the research. Including members from the community in dialog and reflections over decisions concerning their health and wellbeing can improve their quality of life on individual level as well as lead to social sustainability and increased community capacity^39,40^. When community members and researchers work collaboratively to develop interventions, community members themselves can help identify and overcome potential barriers that otherwise remain hidden in traditional interventions designed or proposed by academicians or policy makers. Further, CBPR interventions comprises also of some element of social activities familiar to the community members, making it more accessible and easier for them to incorporate it to their daily routine^38^. A CBPR partnership often also includes other stakeholders from the local public, private and non-profit sectors who are also active during the development of interventions. Through being active partners, the societal actors tend to have an increased understanding about the fruitfulness of the intervention and may thus be more willing to support the future of these interventions even after funding för CBPR research projects end^40^.

Sweden does not have a pre-established initiative within the societal system for working closely with communities nor the knowledge of the needs of communities living in disadvantaged neighbourhoods. Thus, there is a need for working with newer models working closely with the communities to better understand their needs which may be specific to their context. One such initiative is a CBPR programme namely Equal Health that was developed together with the citizens from a socially disadvantaged neighbourhood in Southern Sweden^41^.

### Equal health programme

The program was influenced by Frerian ideologies^42^ and had the aim to create means to reduce health disparities and improve health among citizens in the neighbourhood through participatory and co-operative strategies. This programme started with a trust building phase, where the research team participated in the local activities happening in the municipality meeting places in the neighbourhood to familiarize themselves among the citizens and members of the local women’s network. Following this, the researcher team with the support of the field workers from the municipality, invited citizens from the neighbourhood to participate in explorative future workshops in the neighbourhood^43^. The future workshops gave the opportunity for citizens from the disadvantaged neighbourhood to express their needs and identified strategies and the resources needed to promote health, well-being and quality of life^41^. Following this, the citizens together with the academic partners and other stakeholders engaged in a CBPR planning based on the CBPR model as described by Wallerstein et al.^44^ which led to the establishment of six health promoting living labs corresponding to the problem areas raised during the future workshops namely mental health, oral health and diet, physical activity, social health, safety in the area and women’s health^41^.

The living labs were coordinated by selected members from the community known as lay health promoters (LHP) who had the role of assisting the research team in identifying participants, language interpretation and most importantly acting as bridges for communicating cultural nuances of the community to the research team. The LHPs were schooled in Freirean ideologies of liberation^42^ and trained in participatory methods in specific to manage power mechanisms. They also received support from the research team and social workers from the municipality to handle power imbalances they experienced while facilitating activities in the living labs and recruiting members of the community to the activities. At the structural level they also balanced power when working with stakeholders in the programme to ensure the community members’ voices were not overridden by other actors.

This study describes the evaluation of the CBPR physical activity (PA) intervention implemented within the physical activity lab. The PA intervention was co-developed together with the citizens of the neighbourhood and tested for its feasibility with fifteen participants who participated in the development of the intervention. The development and initial assessment have been presented earlier in the feasibility study by the research team^45^. The feasibility study assessed health-related quality of life, behavioral change and common health parameters as means to monitor change in health, as suggested by the citizens.

Although the feasibility study showed improved health, quality of life and behaviour it lacked clarity on the compliance to the intervention over a longer period since the perceptions relating to health-related quality of life and some of the biomedical parameters were assessed only once post-intervention. Secondly, the feasibility study included only some of the citizens who were involved with the development of the intervention. Thus, there was a need to implement and evaluate the intervention among other citizens from the neighbourhood.

For over two decades it has been repeatedly proven that CBPR health promotions empower communities and contributes significantly to public health efforts in reducing health disparities globally^39,44,46–48^. Several CBPR initiatives specifically targeting physical interventions do exist^49–51^ including some others that were initiated during the pandemic^52,53^. But interventions implemented ahead of the pandemic followed through the pandemic are relatively sparse given that most parts of the world were on lockdown. One CBPR intervention from United States^53^ which was implemented during the pandemic, did not include actual physical training but only motivational dialogues with reflection sessions. In addition, the goals for physical activity were pre-determined by the research team and were virtually evaluated (through online data collection) only during two timepoints before and after the intervention.

Given that the current study was based in the Swedish context where no strict lockdowns were implemented there was a possibility to evaluate the intervention both before and during an ongoing pandemic. Thus, the results of our study may add important knowledge to public health practioners globally regarding the value of establishing CBPR partnerships with communities ahead in time in preparation for future crisis situations.

Thus, the aim of the current study was to evaluate the impact of a CBPR informed physical activity intervention on the health and related behaviours of women living in socially disadvantaged neighbourhoods in Southern Sweden before and during COVID-19.

## Methods

### Context

The intervention programme being evaluated was based in one of the disadvantaged neighbourhoods located in Southern Sweden which was enlisted as highly vulnerable by the Swedish national security agency owing to problems, such as high rate of criminality, low education levels and unemployment^54^. The citizens living in this neighbourhood are non-Swedish speaking migrants predominantly from the Middle east as well as, other Arabic speaking countries^45^.

### Study design

**Figure 1:**
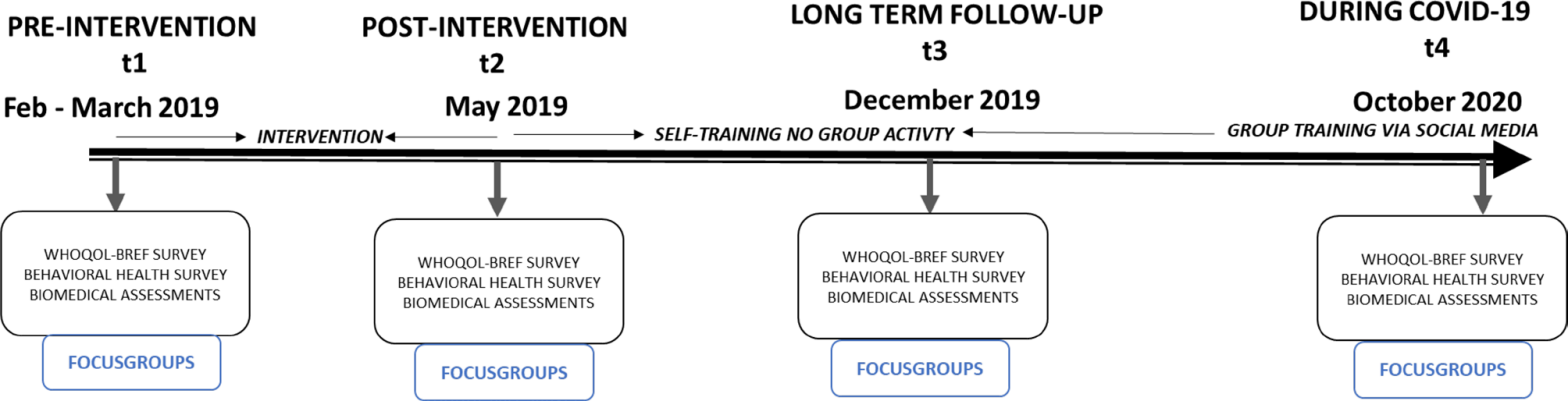
The timeline of events in this study and the qualitative study conducted in parallel.

The current study used a longitudinal evaluation design to assess the health effects of the participatory research informed intervention. Since the current research was based on the community-based participatory research approach the evaluation was an iterative and ongoing process^55^. Quantitative data in form of surveys and biomedical assessments were gathered at four points of time, at first ahead of the intervention (t1), the second measure was precisely after the intervention (t2), the third about six months post-intervention (t3) and the last (t4) during COVID-19 about additional 12 months after t3 (see Fig. 1). The COVID-19 pandemic was not planned from the beginning when the initial data collection was planned. But it was not extended either since the longitudinal evaluation over time was already planned from the beginning within the larger project Equal Health. In contrast to traditional research studies where the design is preset, in CBPR studies the study design is much more flexible as it accommodates an iterative research process. Participants or the community input is considered throughout the research process. In the case of this study there was a collective decision in collaboration with the participants to include the COVID-19 aspect into study aims.

Participants were also engaged in focus group discussion in reflective dialogues about how the intervention and the collaboration with the research team worked during the same four timepoints. However, given the richness of both the qualitative and quantitative data assimilated the authors decided to present them as two different manuscripts so results from both parts are sufficiently reflected rather than condensing them by compromising one for the other. The qualitative study reporting the participants perspectives relating to participation in the CBPR intervention has been previously published by the authors of this manuscript^56^.

### Participants

Three lay health promoters were involved in participant recruitment for this study. These lay health promoters initially reached out to about 40 individuals from the neighbourhood who were not part of the development of this physical activity intervention^45^, but had participated in the initial future workshops^41^. Participants were contacted through face-to-face interactions, as well as, using flyers in Arabic, Swedish and English. There were no specific inclusion criteria for this study as the entire platform was grounded in the participatory principles, which insists on social inclusion. However, in line with the perceptions of citizens that emerged from the feasibility study^45^ the current intervention targeted to include as many women as possible. The research team only ensured if participants had any medical or physical restrictions that could hinder their participation.

### CBPR physical activity intervention

#### Development

The citizens from the neighbourhood who initially raised physical inactivity as a problem during the future workshops together with one of the LHPs co-developed a unique physical activity intervention. The LHP was a was a physical activity enthusiast, born and raised in the neighbourhood. More details about the intervention have been presented in the feasibility study^57^. The intervention consisted of four key aspects namely (1) natural human movements focusing on simple body movements; (2) nutrition and health focusing on healthy diet; (3) restoration and healing included discussion on stress and recovery. The fourth aspect in the intervention was reflection and dialogue where focus was on the importance of physical activity and why it should be accessible for all members in the neighbourhood without differences. The training exercises began with simple body movements to gradually facilitate a change in the citizens’ lifestyle. Over time the activity was designed to gradually turn into more complex exercises customised to individual abilities.

The citizens taking part in the intervention were certified as health ambassadors who could further spread the intervention to others in the community. As health ambassadors the participants could spread their knowledge to others in their family and in their neighbourhood. Such a method has previously been proven effective in spreading the intervention to larger population groups^58,59^. The intervention was offered for free with an intention to make it accessible to all.

#### Feasibility test

The results of the feasibility study with 15 participants identified the need for activities separately for men and women and an immediate need to focus on women as they were isolated and suffered with mental health related issues. The uptake of interventions was perceived better when offered as a group activity and participants also appreciated the possibility to assess their physical health using biomedical tests as they believed that it gave them an evidence of the health impact of the intervention, which further motivated them to be physically active^45,60^.

#### Implementation of intervention

The intervention was offered twice a week in two different groups with fifteen to twenty persons in each of the two groups. Each session lasted for about two hours over a period of three months between March 2019 and May 2019 (Fig. 1). The intervention included physical activity indoors and outdoors, as well as dialogue and reflection sessions focusing on other health determinants associated with physical activity such as diet and stress. The lay health promoters together with other community members certified as health ambassadors who had participated in the feasibility study and initial development of the intervention facilitated these sessions. A minimum of two facilitators from the lay health promoters or health ambassadors were present on all occasions to help the participants. After the intervention program ended, the participants had the opportunity to train on their own or in self-organized groups of their choice for a period of 6 months until December 2019. During this period, they also had the opportunity to communicate with the lay health promoters via social media. With the emergence of the COVID-19 and the physical distancing recommendations the participants actively sought support from the lay health promoters and reinitiated online physical activity sessions as an adapted version of the intervention for the groups.

### Assessments

#### Quality of life and behavioural health surveys

Data was collected using a survey evaluating the different domains influencing health related quality of life using the WHO Quality of Life-BREF survey which is a modified version of the WHOQOL-100^61^. The survey was distributed by the research team together with the health promoter at all four points of time, first before the intervention start at a point t1, the second immediately after the intervention program ended (t2), the third about 6 months after the intervention program ended (t3) and the fourth during COVID-19 pandemic (t4). The survey includes 26 items, of which 24 items were grouped into domains namely physical health, psychological health, social relationships, and environmental domains. The remaining two items were known as global items^61^. The domain scores were calculated from the responses and were later transformed into a 0–100 point scale according to the guidelines of the WHOQOL-BREF^61^. A higher score indicates better quality of life. The questionnaire was available in several languages including Arabic and Swedish. Since most of the participants in this study could only understand Arabic, the Arabic survey was uniformly distributed to the all the participants. The health promoter assisted participants who had difficulties to independently responding to the questions. This survey was previously validated in 15 different locations and the reliability and validity of the Arabic version was also assessed in a similar population^62^. The research team has also previously assessed the psychometric properties of the WHOQOL-Bref survey in the current study context using Rasch models^63^. The results of the psychometric evaluations indicates that all WHOQOL-Bref domains except Social relationships displayed a separation index ≥ 1.5^64,65^ which corresponds to a cronbach α of 0.7 indicating an acceptable reliability of all but one subscale^63^.

Aside of quality of life assessments, participants’ demographic details such as age, gender, mother tongue, educational qualifications, current employment status, socio-economic status, as well as behavioural factors such as diet, physical activity levels, leisure activities were also gathered using standard questions from the national public health survey^66^ at the same four points of time when the quality of life assessments were made. The national public health survey has been administered as an annual cross-sectional surveillance of health, lifestyle and living conditions of the Swedish population living in 21 regions around the country since year 2004. The questions assessing behavioral factors were individual items which were part of the national public health survey. The national public health survey is a collection of individual items and not treated as a unidimensional scale and therefore has not been validated psychometrically. In addition, the questions used in this study have even been used in another study among newly arrived migrants and asylum seekers in Sweden^67^.

#### Biomedical assessments

Physical health was measured using non- invasive biomedical devices. Cardiovascular functioning was monitored through assessment of blood pressure and resting heart rate using iHealth Sense Wireless Wrist monitor from iHealth Labs Inc. (Sunnyvale, California, USA) by placing the device on the participants’ wrist. Blood flow rate and stress index were measured using an AngioScan-01 from AngioScan-Electronics (Moscow, Russia). The device was placed on the participants’ right index finger. Oxygen saturation levels, as an indicator for cardiovascular health and also as measure to understand the condition of the lungs given its relevance to COVID-19, were assessed using an iHealth Air-Pulsoximeter from iHealth Labs Inc. Body mass index, fat mass, and muscle mass were assessed using a Tanita Model MC780MA. The participants’ height was manually entered into the Tanita machine, which further calculated the Body mass index based on the weight measured. Body composition was determined to evaluate the risk for metabolic diseases.

All biomedical assessments were completed in about an hour for each patient on each of the four occasions. A biomedical researcher (SC) who was also part of the research team assisted by lay health promoters performed the assessments. The biomedical experts followed the necessary safety measures particularly during the last occasion during COVID-19, including good hand hygiene and used gloves and facemasks while performing the test in accordance with the recommendation from the Swedish Public Health Agency.

Participants were not requested to follow any protocol ahead of the biomedical tests; however, the participants were tested around the same time of the day at all four-timepoints (see Fig. 1).

### Statistical analysis

Before initiation of the study, the sample size was determined by a power analysis based on the feasibility study^60^. With an alpha set at 0.05 and power at 0.80 approximately, 34 participants were required to identify a detectable change in the quality-of-life scores pre-post intervention.

As a first step in the analysis, all variables, from the biomedical data and the quality-of-life scores, were tested for normality. The non-parametric analysis was preferred over parametric analysis due to the ordinal data in some of the outcome variables. As a second step. Friedman’s test was used to assess differences between the four time points for the quality of life, behavioral and biomedical measures. The Friedman’s test was considered as it is the non-parametric version of ANOVA and is particularly useful given the longitudinal nature of data collected in the study. When the Friedman’s test was significant, the Wilcoxon-signed rank tests was then applied to ascertain differences between specific time points of interest for this study. Such an approach was chosen as an alternative to Friedman’s Rank test as Friedman’s mean rank test relies on the pool of algorithms that were originally included in the study^68^, which means that the differences that happen between t1 and t2 can be compromised by the inclusion of t3 and t4 or vice versa, not allowing to account for other circumstances surrounding these time points such as the COVID-19 pandemic or the absence of an organised intervention.

The scores of the four quality of life domains and two global items, the behavioural factors, and biomedical measures were compared in three stages, viz. measures at t1 prior to invention start *vs*. that of t2 measured directly at the end of the intervention, followed by t2 vs t3 measured 6 months after completion of the intervention and, finally, t3 *vs.* t4 during COVID-19. The comparison of medians were done using Wilcoxon’s Signed Rank Test models^69^. The changes were monitored at group level. Statistical significance was set at a* p* value of < 0.05. The p-values of the Wilcoxon-signed rank tests were then adjusted using the Bonferroni correction based on the total number of comparisons being performed. Effect sizes were finally calculated and interpreted based on reference levels, *d* = 0.2 or lower was considered small, 0.2 < *d* < 0.5 as medium, and *d* = 0.8 or larger as large^70^.

### Ethical considerations

The lay health promoters verbally informed all participants through a video call via Whatsapp in Arabic language about the purpose of the research study prior to the first data collection occasion. Participants were also assured that participation was voluntary and that they were allowed to leave the study at any point in time without any consequences.

In the later part during COVID-19 participants were assured that all activities were to be carried out in accordance with guidelines from the Swedish Public Health Agency. Participants were also ensured that the biomedical researchers who collected biomedical data would follow the necessary safety measures, including good hand hygiene in accordance with the Swedish Public Health Agency’s recommendations. All the above information was also provided to the participants in writing together with contact information of the research team. Participants were asked to sign an informed consent form during the first occasion. All data collected were anonymized and kept confidential. The data was only accessible to the members of the research team.

### Ethics and consent

This study was conducted in line with the Helsinki Declaration and Swedish Ethical Review Authority approved the study (DNR 2018/382 and DNR 2020-04063). The data collected are anonymized and securely stored. Participants were given verbal and written information and written informed consent was obtained. They were also informed that participation was voluntary, and they were free to leave the study at any point of time without consequences.

## Results

All participants in this study were of Arabic descent and were aged between 23 and 77 years. The demographic characters of participants are presented in Table 1.

**Table 1:** Demographic characteristics of participants.

Characteristics	Participants n = 34
Age	
Range (md)	23–77 (49)
Country of origin	
Iraq	10 (29%)
Syria	8 (24%)
Lebanon	5 (14%)
Palestine	4 (12%)
Iran	4 (12%)
Algeria	1 (3%)
Egypt	1 (3%)
Sudan	1 (3%)
Educational qualification	
University education	7 (19%)
High school	11 (31%)
Elementary school	16 (50%)
Employment status	
Employed	3 (9%)
Sick leave	2 (6%)
Parental leave	2 (6%)
Studying/internship	10 (29%)
Retired	9 (27%)
Home maker	8 (23%)

Of the 40 females who were invited 34 (88%) of them participated in the intervention programme, and the research study. Three of the forty participants, who were initially invited dropped out ahead of the start of the intervention since the training times did not match their individual and family commitments or schedules. Data from three participants were excluded, as they were not present in two of three test occasions when research data were collected. At the final follow-up during COVID-19 at time point t4 only 28 participants were available, since three of the 35 women who were invited could not participate as they had not returned from their home country after summer vacation due to unavailability of flights. One other woman who dropped out had been diagnosed with COVID-19 2 days ahead of data collection. Three participants mentioned that they feared being infected, as they belonged to a risk group and thus refused to participate. Dropout analyses in terms of baseline behaviour, biomedical parameters and quality of life scores showed that the women who dropped out did not significantly differ from those who remained in the study.

### Health related quality of life

The median and range of the WHOQOL-BREF domain scores at the three points of time is presented in Table 2 together with the Friedman’s coefficient*.* Overall, there were significant changes in the psychological, social and environmental health domains as well as, in the perceived health satisfaction over time. The mean score significantly increased for all of quality of life domains (*p* < 0.05) except the physical health domain (*p* > 0.05) at time point t2 compared to t1. However, there were no remaining significant changes at point t3; rather the perceptions reversed after 6 months of completion of the physical activity intervention at point t3 compared to t2. The participants did however perceive an increased satisfaction in their health at time point t4 as compared to that in t3. There was also an increase in environmental health domain scores (*p* < 0.05) between t3 and t4. The effect sizes for change in perception of health-related quality of life at time point’s t1–t2, as well as t3-t4 were overall low. The results from the health-related quality of life measures is presented in Table 3.

**Table 2:** Descriptive statistics of all variables time points t1–t4 and Friedmans test comparing differences between the time points.

Health and behavioural variables	Friedmans test Χ^2^	t_1_ _Median (range)_ _n = 34_	t_2_ _Median (range)_ _n = 34_	t_3_ _Median (range)_ _n = 34_	t_4_ _Median (range)_ _n = 28_
I. Quality of life domain					
Physical domain	3.2	56 (19–88)	63 (13–94)	56 (19–94)	56 (19–88)
Psychological domain	8.7*	56 (25–81)	69 (25–94)	56 (6–94)	56 (19–94)
Social relationships domain	7.9*	56 (19–100)	75 (44–100)	69 (31–100)	69 (25–100)
Environmental domain	6.5*	56 (19–94)	63 (25–88)	56 (25–88)	63 (38–88)
Quality of life	1.32	4 (1–5)	4 (1–5)	4 (1–5)	3 (2–5)
Health satisfaction	11.5*	3 (1–5)	4 (1–5)	4 (1–5)	4 (1–5)
II. Behavioral factors					
Leisure time physical activity	14.4*	1.0 (0–5)	3.0 (0–5)	2.0 (0–5)	1.0 (0–5)
Everyday activities	21.1*	2.0 (0–6)	4.0 (1–6)	2.0 (0–6)	3.0 (0–6)
Hours of sitting	2.1	5.0 (0–6)	5.0 (0–6)	5.0 (0–6)	5.0 (0–5)
Vegetable intake	16.5*	3.0 (5.0)	5.0 (4.0)	3.0 (0–5)	4.5 (0–5)
Fruit intake	18.9*	4.0 (0–5)	5.0 (2–6)	4.0 (0–6)	5 (0–6)
Fish intake	1.2	2 (1–4)	2 (1–4)	2(0–4)	2 (1–4)
Soda intake	3.6	3.0 (0–4)	3.0 (0–5)	3.0 (0–5)	3.0 (0–4)
III. Biomedical parameters					
Oxygen saturation	4.9	98 (92–99)	98 (94–99)	98 (96–99)	97 (93–99)
Systolic blood pressure	7.9*	126.5 (96–162)	117.5 (98–164)	123 (98–180)	125 (88–196)
Diastolic blood pressure	6.6	80 (55–104)	75 (62–98)	74 (62–98)	77 (45–99)
Resting heart rate	7.4	78 (60–90)	77.5 (60–92)	78 (56–96)	73 (48–84)
Blood flow rate	12.7*	329 (273–371)	334 (295–386)	324 (207–385)	334 (274–395)
Stress index	2.9	199 (25–675)	186 (6–1087)	252 (50–1055)	128 (12–899)
Body mass index	0.4	30.7 (21.7–44.4)	30.5 (20.7–43.7)	30.2 (20.7–40.8)	29.5 (20.0–41.0)
Muscle mass	0.7	46 (34.3–61.0)	46.2 (35.6–62.9)	44.6 (34.0–60.7)	45 (36.0–61)
Fat mass	6.7*	27.2 (14.2–56.7)	26 (12.9–56.6)	28.6 (13.4–50.9)	27.6 (12.0–50.0)

*Adjusted *p*-value < 0.05.

**Table 3:** Wilcoxon signed rank test comparing quality of life domains and that were statistically significant in the Friedman’s test across the four timepoints t1–t4.

Health and behavioural variables	t2–t1 n = 34			t3–t2 n = 34			t4–t3 n = 28		
	Mean difference (SD)	z	Effect size	Mean difference (SD)	z	Effect size	Mean difference (SD)	z	Effect size
I. Quality of life domains									
Psychological domain	10.1 (12.4)	− 3.9*	0.5	− 9.7 (23.6)	− 2.2*	0.3	1.22(16.66)	− 0.36	0.0
Social relationships domain	8.6 (16.9)	− 2.9*	0.3	− 0.9 (23.3)	− 0.2	0.02	− 4.70 (19.93)	− 1.76	0.2
Environmental domain	5.4 (13.3)	− 2.3*	0.3	− 4.9 (12.6)	− 2.1*	0.3	8.04 (16.54)	− 2.26*	0.3
Health satisfaction	0.6 (1.1)	− 3.0*	0.4	− 0.5 (1.4)	− 2.0*	0.2	0.59 (1.15)	− 2.37*	0.3
II. Behavioural factors									
Leisure time physical activity	1.4 (1.7)	− 3.9*	0.5	− 0.7 (1.7)	− 2.2*	0.3	0.8 (2.21)	− 1.53	0.2
Everyday activities activity	1.4 (2.0)	− 3.3*	0.4	− 0.7 (1.7)	− 2.3*	− 0.2	− 1.18 (2.53)	2.29*	0.3
Vegetable intake	1.7 (2.0)	− 4.0*	0.5	− 0.7 (1.5)	− 2.4*	− 0.3	1.03 (1.84)	− 2.66*	0.4
Fruit intake	1.1 (1.7)	− 3.1*	0.4	0.5 (1.4)	− 2.1*	0.3	1.00 (1.70)	− 2.79*	0.4

*t*_*1*_ pre-test, *t*_*2*_ post-test, *t3* 6 months follow-up, *t4* during COVID-19.

*Bonferroni adjusted *p*-value < 0.05.

### Behavioural factors

The descriptive scores for the different behavioural factors at the three points of time is presented in Table 2 together with the Friedman’s coefficient. The physical activity intervention also yielded direct and positive significant changes in behaviour especially concerning nutrition and fitness at t2 compared to t1 (Table 3). After participation in the intervention, the women were significantly more likely to consume more vegetables and fruits (*p* < 0.05) than ahead of their participation in the intervention at all three times compared t–t2, t2–t3 and t3–t4. With regards to fitness behaviour, the results indicated that participants spent significantly more time on physical activity or sports and reported to have a more active lifestyle in their everyday life (*p* < 0.05) at t2 compared to t1. Physical activity levels reversed back at time point t3 compared to t2 but were still higher than t1 while observing the descriptive scores. There was a significant increase in everyday activities and fruit and vegetable intake at point t4 compared to t3 and earlier (*p* < 0.05). The intervention did not significantly influence soda intake, fish intake and hours of sitting at all points of time. The effect sizes for change in behaviour before and after intervention both at t2, t3 and t4 was low to moderate.

### Biomedical assessments

The descriptive measures for the different biomedical assessments at the three points of time are presented in Table 2 together with the Friedman’s coefficients. Systolic blood pressure significantly decreased (*p* < 0.05) at t2 when compared to t1. The levels were also significantly lower at time point t3 compared to t1 (*p* < 0.05). Blood flow rate increased significantly between timepoints t1 to t2 (*p* < 0.05), decreased between timepoint t2–t3 and increased again between t3 and t4 (*p* < 0.05). There was a significant difference in fat mass at time point t2 compared to t1, but the effect significantly reversed at point t3 (*p* < 0.05). Blood flow rate increased significantly between timepoints t3 and t4. All other biomedical parameters assessed remained stable and within normal intervals at timepoints t2, t3 and t4. The results from the biomedical assessments are presented in Table 4.

**Table 4:** Wilcoxon signed rank test comparing biomedical parameters that were statistically significant in the Friedman’s test across the four timepoints t1–t4.

Biomedical parameters	t2–t1					t3–t2			t4–t3			
	N	Mean difference (SD)	z	Effect size	N	Mean difference (SD)	z	Effect size	N	Mean difference (SD)	z	Effect size
Systolic blood pressure	32	− 8.2 (14.3)	− 2.9*	− 0.4	31	2.4 (17.9)	− 0.9*	− 0.1	26	1.58 (12.19)	− 0.73	0.1
Blood flow rate	32	12.9 (27.8)	− 2.2*	− 0.3	25	− 19 (44)	− 2.5*	− 0.4	26	− 15.8 (1.57)	− 2.66*	0.4
Fat mass	31	− 0.6 (1.0)	− 2.7*	− 0.3	32	1.4 (5.9)	− 2.1*	− 0.3	26	− 1.23 (6.54)	− 0.17	0.0

*t*_*1*_ pre-test, *t*_*2*_ post-test, *t3* 6 months follow-up, *t4* during COVID-19.

*Bonferroni adjusted *p*-value < 0.05.

## Discussion

The intervention programme seems to have succeeded in increasing the daily physical activity levels of women from disadvantaged neighbourhoods. The participants in this study perceived a higher health related quality of life directly after participation in the intervention program, but the perceived quality of life reversed 6 months after the organized intervention ended. However, it was also noted that during the pandemic the participants experienced no changes in their quality of life. Moreover, the participants seemed to have continued to engage themselves in activity even during the follow-up period (despite the absence of organized activities) as well as during the pandemic. Similar results were also observed regarding the consumption of fruits and vegetables. These behavioural changes were partly reflected on the physical health since there was a decrease in the levels of systolic blood pressure, 6-month post intervention. Thus, this study seems to have some evidence indicating that the participatory research informed physical activity intervention programme had a potential impact on some of the important markers of cardiovascular disease both in terms of changes in risk behaviour as well as physical and mental health thereby assisting in reducing health disparities.

This study also shows that an increased score in the environmental health domain after participation in the intervention indicating an increased trust in the health care system and other public agencies compared to that ahead of the intervention. The environmental health domain from the WHOQOL-BREF survey measured seven different environmental/policy related factors which included satisfaction in health care. On examining the raw scores of the individual items, satisfaction with access to health care was seen to have considerably increased at t4 compared to other items. This finding was further confirmed during the focus group discussions with the participants, who reported that through the support received from the lay health promoters with whom they interacted through the Whatsapp group they gained better understanding of the recommendations and health information distributed by the health and social care and thus began to have more faith in the systems^56^.

The results of the current study also demonstrates that participation in the physical activity intervention program led to sustainable increase in habitual physical activity levels among women in the community who otherwise reported to have led a sedentary lifestyle directly after intervention. Women also became conscious about their diet and began consuming more vegetables and fruits after taking part in the intervention. The change in physical activity levels together with dietary changes were also reflected on physical health of the participants with significant improvement in at least one of the risk markers for chronic diseases, i.e., blood pressure, directly after participation in the intervention.

As seen elsewhere in the world^23,71^, this study also showed a reduction in organised physical activity during COVID-19 pandemic. However, women seemed to have identified alternate means to be active for example there was a significant increase in everyday activities such as climbing up and down the stairs indoors, cycling and walking in order to compensate the absence of organised physical activity. This was also reconfirmed from the focus group discussions with the women by the research team. Some of the women also reported that they were depressed when they could not engage in physical activities during the pandemic despite being aware that it was important to preserve health during the pandemic. So the women said they tried to increase their everyday activities to compensate for the exercises they performed during the pandemic^56^.

Interestingly in the current study participants had increased their frequency of engagement in everyday activities when they were unable to perform physical exercise or sports in public premises due to the COVID-19 pandemic. These findings were consistent with the discussions held with the women where they reported that they felt frustrated and guilty if they could not maintain a physically active lifestyle. This was also prominent during COVID-19 and thus, they found alternate means to keep themselves physically active including performing more everyday activities and using the stairs instead of the elevator^56^.

Although measures on everyday activities increased significantly during the pandemic (t4) compared to that at time-point t3, there was still a decrease in everyday activities at t4 compared to that post-intervention (t2). A previous study among migrant women particularly those who migrated from warmer countries in the middle east to Sweden showed that leisure time activity such as walking, or cycling decreased significantly during winter season^72^. In the current study, the intervention was facilitated during late winter and beginning of spring in Sweden and the t3 and t4 measures happened during winter. This may also explain the decrease in everyday activity during long-term follow up. In addition, the discussions with the women also revealed that they were usually depressed during winter as it was dark and cold, so they wanted to have more organised physical activities during these times as training in groups helped them recover from depression^56^.

The results relating to objective physical health assessed by biomedical tests after the intervention in the current study are also in line with a randomized controlled trial evaluating a culturally adaptive physical activity intervention offered by the primary care to migrants with high risk for cardiovascular disease^73^. The intervention evaluated in the previous study was however, not informed by participatory research. An earlier review study assessing the impact of interventions targeting health related behaviours also suggest that actively working with health literacy and knowledge provision helps promote health and change in behaviour among community members^74^.

The continued engagement in physical activity among women during the period of intervention in the current study may be attributed to the participatory nature, where the women themselves actively participated in the research process and set their priorities based on their needs, while other stakeholders and academicians facilitated the knowledge transfer process together with the women in a power neutral environment in an attempt to foster empowerment which may in turn be a means to long lasting behavioural change^75^. In addition, participants also received more information about the biomedical assessments and their own results, which they frequently missed during their visits to the primary care according to results from the feasibility study^45^.

The results from the focus group discussions with the participants of this study^56^ revealed that women took initiative to reconnect with the other women in the group through social media during the pandemic. The women received social support from each other which provided them strength to revive and also reiniate the group physical activities with the support of the lay health promoters. This in turn seems to have helped them maintain their physiscal health even during an acute situation such as the COVID-19. This was further strengthened by the results from the current study where despite the rapidly spreading pandemic the women had reported an increased satisfaction in their own health. These results were much in contrary to other studies^24,76–78^ which showed that lifestyle had worsened among citizens living in disadvantaged situations while they also percieved poor health and symptoms related to mental ill-health during the pandemic thus unveiling the potential effect of a participatory physical activity intervention on behaviour and health of women in disadvantaged neighbourhoods. Such a social support may have an impact in not only maintaining health during the pandemic but also introducing community resilience given the evidence from prior research which shows that prolonged engagement in neighbourhood activities in groups creates a feeling of social connectedness^79,80^.

Despite that the participants became certified as health ambassadors and had even created their own training groups with other women from the neighbourhood during the long-term follow up, the findings from the focus group discussions suggested that they experienced challenges to train with their own groups. This was owing to lack of access to indoor facilities and inability to independently coordinate practical aspects such as scheduling times and holding contact with their groups as that done by the lay health promoters^56^. Thus, the role of the lay health promoters may also be of interest in this study since together with facilitating activities they have also been instrumental in bridging between participants and creating a bond between them leading to the formation of a social support network. This was further confirmed in the focus group discussions where the participants reported that the lay health promoters had suported them individually to help address their challenges and uncertainties through bringing them to a social context which helped them identify their own strengths^56^. Aspects such as social support through participation in social networks relate to the concept of social capital, which is the ability of individuals to acquire mutual benefits through being members in certain social networks. This may relate to the case of the current study where performing physical training as a group may be associated with changes in perceived health acquired with the support of the social network^81,82^.

## Limitations

Although the results from this study show both individual and societal benefits from participating in the intervention, there were also some methological limitations. One key limitation in this study is self-selection. Further, the health status of the participants and the medications they may have consumed can be regarded as potential confounders in this study. No information regarding the participants medical history was assimilated in this study as well as the qualitative study. However, the discussions with the participants during focus groups have been elaborated in the discussion for the purpose of triangulating and reconfirming our findings. In addition, the lack of a control group, limits the possibility to elaborate on the causal effects of the current findings, as well as generalization of the findings to a larger population/community. Although the statistical significance may be affected by these biases, CBPR studies often place ethical aspects of inclusion and equity more central than optimal research designs with randomisation and controls (or RCTs). RCT measures causal relationships but not elucidate the mechanisms of change and the interactions that happen with precision. Further, RCTs emerges from the assumption that complex mechanism can be measured by breaking into smaller components which can be independently measured. However, CBPR studies focuses on situated knowledge in a particular population group that promotes active learning from the specific context as a whole. Thus, measuring fragments may not elucidate the utility of CBPR interventions. Further the impact of the CBPR interventions are assessed both based on process and outcomes which are defined by the community members themselves. Designing an RCT study where the participants themselves have been part of designing the intervention and evaluation may not often be feasible. Therefore, prior research recommends assessing the impact of CBPR studies using a wide range of outcome measures^83,84^ as done in this study. Future studies with larger and more diverse samples (to also allow for subgroup analysis) and a better controlling design (with control groups and/or cross-over designs) could provide more and better evidence in relation to the intervention program.

The results from the biomedical test should also be interpreted with caution since information relating to medications consumed by the participants were not obtained and could therefore influence the variables measured. Another limitation is missing data in the biomedical parameters’ due to technical issues faced while the biomedical assessments were performed. Further, the validity of the results obtained from the non-invasive testing over the more standard invasive measures may be seen as limitation. The novel non-invasive techniques used in this study^45^ have however previously shown to be a stress-free and less time consuming alternative to invasive measures. Including only women can be considered as another potential generalization limitation in this study. However, the learnings from the feasibility study indicated that women in these neighbourhoods are often isolated and prone to lifestyle related health problems compared to their male counterparts. In addition, the men in the neighbourhood had access to several other forms of physical training including gyms and outdoor training facilities unlike women who had traditional restrictions to train in public or together with other men^45^.

Although some of the conclusions of the causality between the programme and the outcomes can be biased by other factors as well. It must be noted that the women themselves have said during focus group discussions that this intervention was meaningful unlike other activities available in the neighbourhood in that it was not only adapted to their needs, but that they themselves were able to decide and reflect together regarding the intervention which was also the reason for its uptake^85^.

## Conclusion

This participatory research informed physical activity intervention program shows some beneficial impact on both physical health and behaviour in women from disadvantaged backgrounds and in addition paved way to increasing resilience during the pandemic. Building on existing CBPR programs may be an effective means to enhance community resilience in disadvantaged neighbourhoods even during an ongoing pandemic. Considering the dual burden of the two pandemics and its association to chronic diseases future efforts should target building on existing participatory programs to enhance pandemic preparedness and preserve health among citizens living in disadvantaged neighbourhoods and thereby contribute to efforts towards reducing health disparities.

## Data Availability

The datasets used and analysed during the current study are available from the corresponding author on reasonable request.
